# Effect of Different Harvest Time and Microwave Aging on Aroma Characteristics of Japanese Apricot Wine

**DOI:** 10.3390/molecules30020392

**Published:** 2025-01-18

**Authors:** Aoting Li, Jiangfeng Song, Yike Wang, Xiao Huang, Silas Segbo, Zhihong Gao

**Affiliations:** 1 College of Horticulture, Nanjing Agricultural University, Nanjing 210095, China; 2022804141@stu.njau.edu.cn (A.L.); 2022804246@stu.njau.edu.cn (Y.W.); huangxiao@njau.edu.cn (X.H.); silsegbo@gmail.com (S.S.); 2 Institute of Agro-Product Processing, Jiangsu Academy of Agricultural Sciences, Nanjing 210014, China; songjiangfeng102@163.com

**Keywords:** Japanese apricot, steeped mume wine, aroma component, nutritional quality, microwave aging

## Abstract

The aroma and nutrition of Japanese apricot fruit change continuously as the fruit ripens. The differences in fruit aroma and nutrition can affect the resulting wine, which is produced by steeping the Japanese apricot fruit. In this study, we used HS-SPME-GC-MS to examine the aromatic compositions of Japanese apricot fruit and wine produced from its macerated fruit at different levels of ripeness. We employed UPLC to examine the nutritional components such as organic acids, amygdalin, and phenolics to support the identification of the optimal ripening time. The microwave aging technology was also used to treat the steeped mume wine in order to explore the optimal conditions for microwave aging. We found that the optimum aroma period for the ‘Nannong Longxia’ fruit was 81 d after flowering. Furthermore, the changes in the aroma of mume wine after steeping in 65%vol base wine were closer to those of the fruit than those of 42%vol base wine, and the optimum period for the aroma of mume wine after steeping was the same as the fruit. Analysis showed that the optimum nutritional period for the fruit coincided with the optimum aromatic period. The best aromatic and nutritional components of the finished wine were obtained when the fruit was picked 81 days after flowering and the steeped mume wine was made with a 65%vol base wine. Microwave aging technology can significantly increase the proportion of esters in the aroma composition and reduce the content of acids. Among them, 500 W treatment at 50 °C for 80 min had the best effect on improving aroma components. These findings represent a theoretical foundation for exploring the aroma optimum period for Japanese apricot fruit and steeped mume wine, and for determining the optimum harvest periods for production.

## 1. Introduction

Japanese apricot fruit (*Prunus mume Siebold & Zucc.*) originates from China, and its processed product, steeped mume wine (mume wine), has been handed down to generations [1]. Mume wine is categorized into two distinct types based on production methods: steeped mume wine and fermenting mume wine. Steeped mume wine is derived from the direct maceration of Japanese apricot fruit in base wine, while fermenting wine is produced through the fermentation of Japanese apricot fruit by yeast. Notably, the steeped method is favored for its ability to retain the fruit’s aroma, and this approach is employed in the present study. Steeped mume wine has many nutrient contents benefiting human health as long as drinking is moderate. The citric acid, which accounts for about 90% of the total organic acids in steeped mume wine [2], is an important intermediate product in the tricarboxylic acid cycle reaction and helps the body with nutrient absorption, glucose metabolism, and antibacterial and anti-inflammatory functions [3,4]. Phenolics, known for their potent antioxidant properties, serve as natural scavengers that effectively neutralize oxygen-free radicals within the human body [5]. Amygdalin is widely found in the seeds of plants of the Rosaceae family [6], and is commonly used in the medical field as an expectorant [7], cough suppressant [8], and adjuvant anticancer drug [9]. However, the presence of excessive amygdalin levels in steeped mume wine poses a significant risk to human health, with potentially fatal outcomes in severe cases. Consequently, it is imperative to accurately quantify the amygdalin content within steeped mume wine.

In addition to nutrient components, steeped mume wine also has a unique aroma. Through unremitting research by generations of researchers, hexyl acetate, benzaldehyde, 2-hexenal, and linalool were proved to contribute greatly to the aroma components of the wine [10,11]. However, the contents of the aroma and nutrients in different fruit ripening periods of Japanese apricot fruit have not been extensively studied. In addition, accelerated aging treatment has been a very skilled technology in the fruit wine processing industry, which can make the wine rapidly age in a short period of time, make physical and chemical properties meet the standard of finished products [12], and reduce time and capital costs. The most widely used artificial aging methods currently include ultra-high-pressure aging, ultrasonic aging, microwave aging, and so on. The microwave aging method, which we focused on in this research, has the advantages of accelerating the maturity of wine color, promoting esterification reaction, and improving sensory evaluation [13]. The primary aim of this study is to detect the aroma and nutrient composition changes in Japanese apricot fruit and steeped mume wine at different ripening stages. Secondly, it focuses on how to accelerate the aging of the best steeped mume wine; identify its aroma; and finally, find the best fruit harvesting time and maceration method of wine. This study delineates the optimal harvest timing for processed Japanese apricot fruit varieties, examines the aromatic and nutritional profiles of steeped mume wine, and explores its aging techniques. The findings offer valuable insights into the Japanese apricot wine processing industry.

## 2. Results and Analysis

### 2.1. Color Variations in the Maceration Process of Steeped Mume Wine

**Figure 1:**
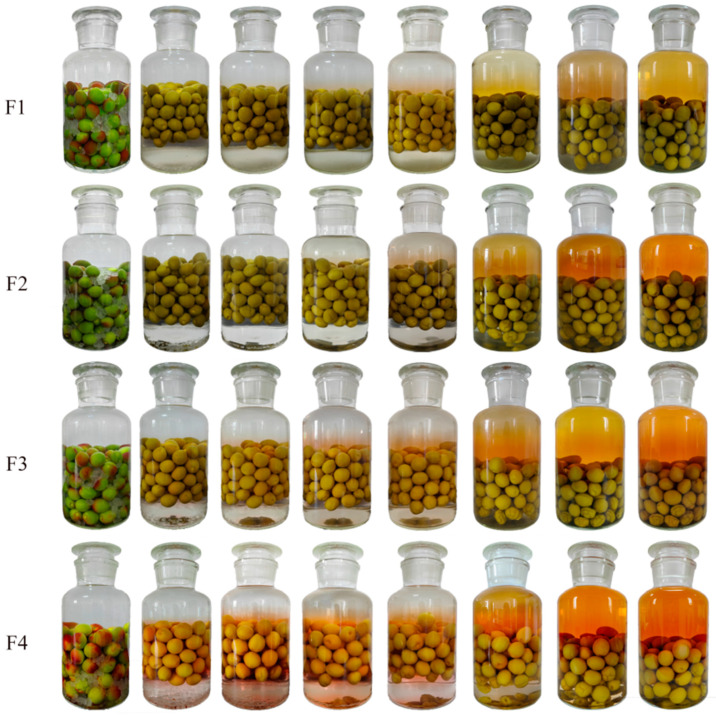
Color variation of fruit-steeped mume wine made from 42%vol base wine. Note: the pictures were taken from left to right after 1 day, 1 week, 2 weeks, 3 weeks, 4 weeks, 8 weeks, 12 weeks, and 14 weeks of maceration. The meaning of F1–F4 see Table 3.

**Figure 2:**
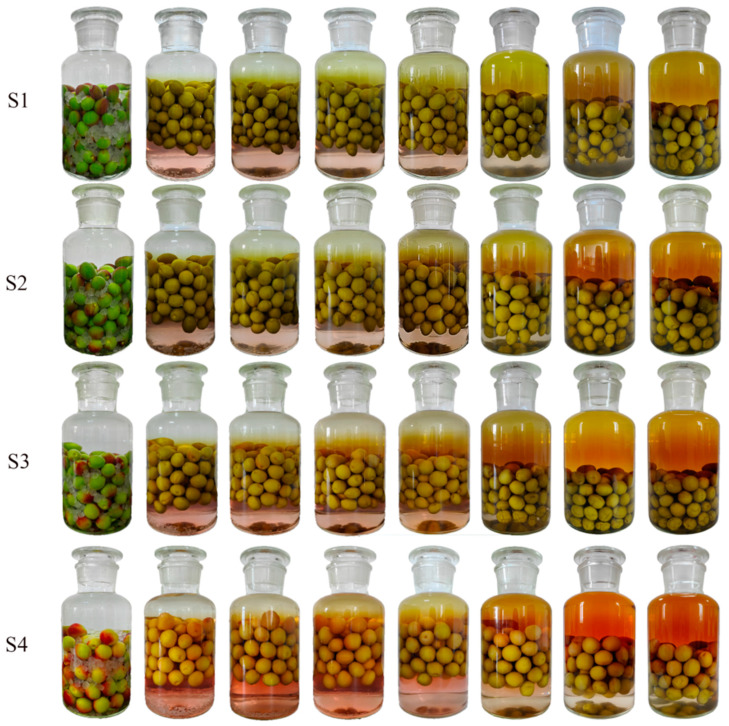
Color variation of fruit-steeped mume wine made from 65%vol base wine. Note: the pictures were taken from left to right on the first day after 1 day, 1 week, 2 weeks, 3 weeks, 4 weeks, 8 weeks, 12 weeks, and 14 weeks of maceration. The meaning of S1–S4 see Table 3.

Figure 1 and Figure 2 show that fruit maturity affects the final color of mume wine. The wine made from less ripe fruit appears yellow–green, while the mume wine from more mature fruit turns orange–red. Additionally, the strength of the base wine slightly influences the wine’s color, with stronger base wine resulting in a darker shade. During maceration, the peel of Japanese apricots fruits loses its original emerald green hue, becoming dull over time. After one week of maceration, the fruits begin to float in the middle of the bottle. Over the course of three months, they gradually sink back to the bottom. The timing of color change in the steeped mume wine depends on both the fruit’s maturity and the base wine’s strength. As the fruit ripens more and the base wine becomes stronger, the color of the wine appears earlier in the process.

### 2.2. The Effect of Fruit Ripeness on the Volatile Components of Fruit and Steeped Mume Wine

**Figure 3:**
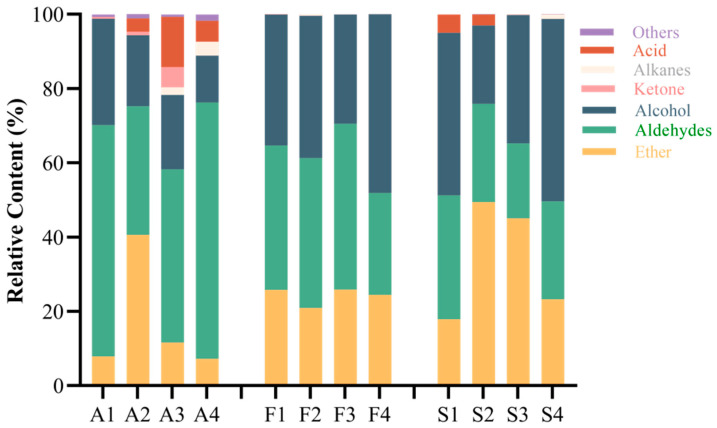
Percentage of volatile components in Japanese apricot fruit and steeped mume wine. Note: percentage of volatile components in Japanese apricot fruit (A1–A4), steeped mume wine made from 42%vol base wine (F1–F4), and steeped mume wine made from 65%vol base wine (S1–S4).

The increase in ester content indicates the change in fruit from green to ripe. As can be seen in Figure 3, the ester content showed a mountainous peak during fruit development. The highest content of esters accounted for 40.6% of the total volatilization. At this time, the main contents of the fruit included propyl acetate, ethyl butyrate, hexyl acetate, ethyl caproate, methyl benzoate, etc. When the content of esters increased, the content of aldehydes decreased in fruit. The content of aldehydes was the lowest in A2, only hexal and 2-hexenal. The contents of aldehydes are higher in A1 and A4. The contents of hexal, 2-hexenal, and chlorophylla aldehyde are higher in A1, while the contents of pentylaldehyde and hexal are higher in A4. At A3, the contents of acetic acid and acetone increased, resulting in an increased proportion of ketones and acids in fruits. At the same time, the amount of n-pentane in the fruit began to increase and continued to A4. Alcohols were abundant in all four periods, mainly ethanol and (E)-2-hexene-1-alcohol.

The volatile components of F1–F4 did not change greatly with the change in fruit maturity. The content of esters accounted for 20~30% of the total volatile components. The content of aldehydes was stable at about 40% in the early stage and dropped to 27.4% after F3. The proportion of esters in S1–S4 was similar to that in A1–A4. The content of esters was the highest in S2, which mainly included ethyl acetate, ethyl butyrate, ethyl 3-methylvalerate, propyl lactate, etc. The content of aldehydes also maintained the characteristics of the fruit, which was relatively high in S1 and S4 and relatively low in S2 and S3. It is worth mentioning that the acid content of S1 and S2 substances was high, mainly pentanoic acid and a small amount of butanoic acid. With the increase in fruit ripeness, the content of acids in the impregnated wine decreased, opposite to the fruit (See annexed Tables S1–S3 for more aroma components data).

### 2.3. Effect of Different Fruit Development Periods on the Volatile Component of Fruit and Steeped Mume Wine

**Figure 4:**
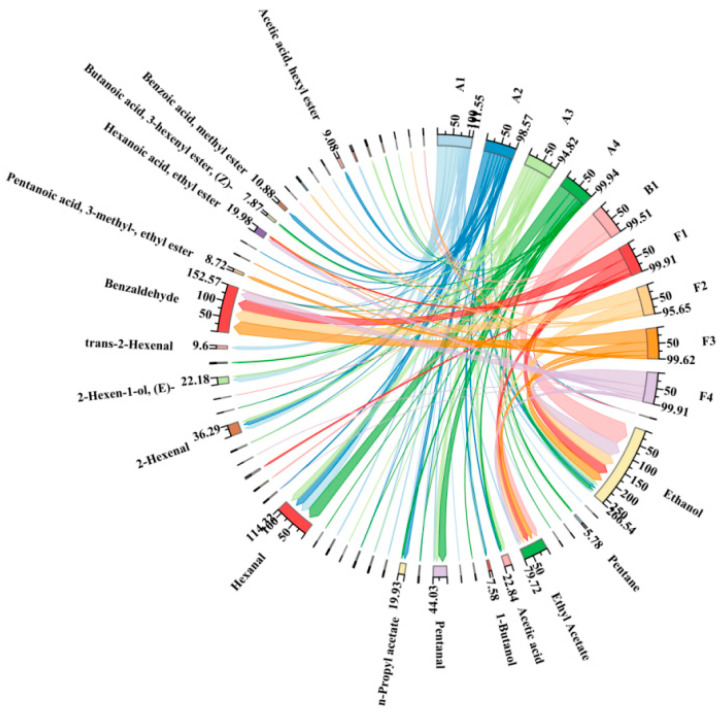
Choropleth of aroma composition in Japanese apricot fruit (A1–A4), 42%vol base wine (B1), and steeped mume wine made from 42%vol base wine (F1–F4). Note: the compounds with less than 0.3% have no name shown.

**Figure 5:**
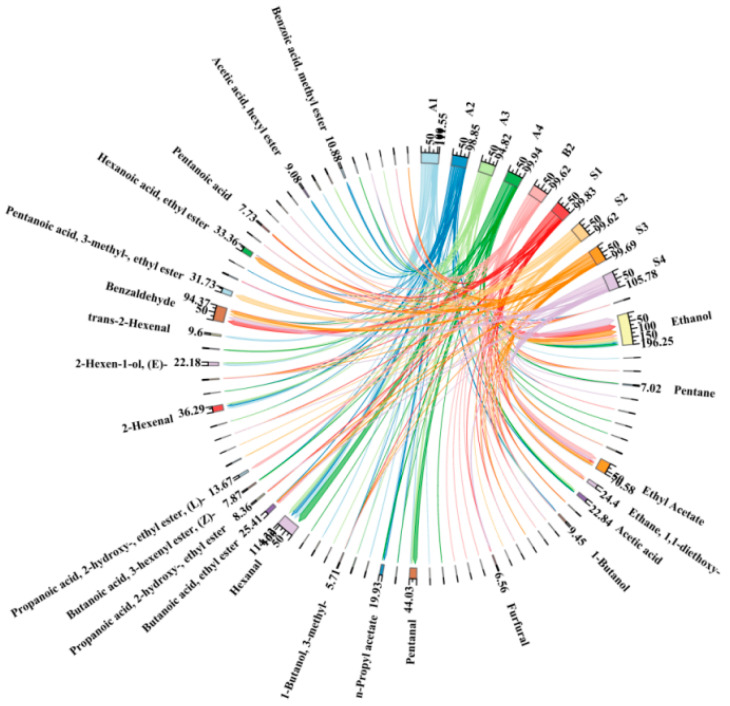
Choropleth of aroma composition in Japanese apricot fruit (A1–A4), 65%vol base wine (B2), and steeped mume wine made from 65%vol base wine (S1–S4). Note: the compounds with less than 0.3% have no name shown.

In Figure 4, ethanol was present in all the Japanese apricot fruit, the 42%vol base wine (B1), and the steeped mume wine (F1–F4), although the amount of ethanol in the fruit was much smaller than in the 42%vol base wine and the steeped mume wine. Hexanal was present in large quantities in the fruit during the developmental period, and its levels decreased and then increased. Benzaldehyde was the most abundant constituent in F1–F4, and ethyl acetate was much more abundant than in the fruit. Figure 5 shows more aroma components than Figure 4, and the number of lines in the figure is also higher, indicating that more aroma components can be produced with 65%vol base wine. The cases of ethanol, hexanal, benzaldehyde, and ethyl acetate in the graph were consistent with the above, with the difference that there are significantly more ethyl esters in S1–S4 than in F1–F4, mainly 1-hexanoic acid, butyl ester, butanoic acid, 3-hexenyl ester, (Z)-, pentanoic acid, 3-methyl-, ethyl ester, and so on.

### 2.4. Changes in Aroma Substances During the Production of Steeped Mume Wine

**Figure 6:**
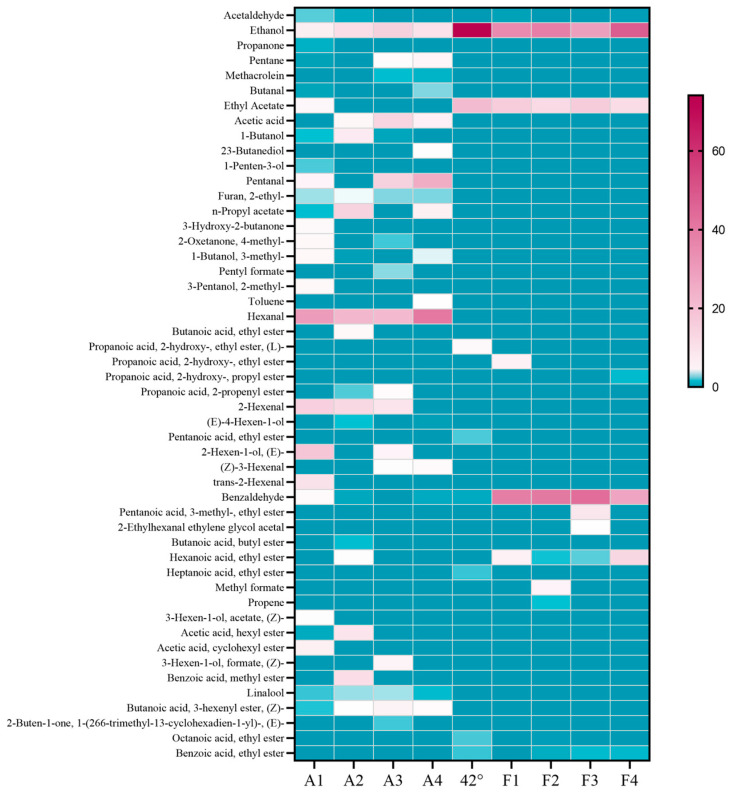
Heat map of aroma composition in Japanese apricot fruit (A1–A4), 42%vol base wine, and steeped mume wine made from 42%vol base wine (F1–F4).

From 71 volatile components in the fruit, 42%vol base wine, and macerated wine made from 42%vol base wine, 50 representative substances with apparent variations were selected to make a thermogram, as shown in Figure 6. It can be seen that the main playful substances in the 42%vol base wine are ethanol, ethyl acetate, and ethyl lactate, which account for about 88% of the total volatiles. The substance with the highest content percentage within the fruit is hexanal, while 2-hexenal and pentanal are also high in the fruit. Aldehydes are the main components of the aroma in ‘Nannong Longxia’ fruit. Other substances with higher content were ethanol, (E)-4-hexen-1-ol, acetic acid, propyl acetate, and methyl benzoate. The three components with the highest content percentage in steeped mume wine were ethanol, ethyl acetate, and benzaldehyde(See annexed Table S1 and Table S4 for more aroma components data).

**Figure 7:**
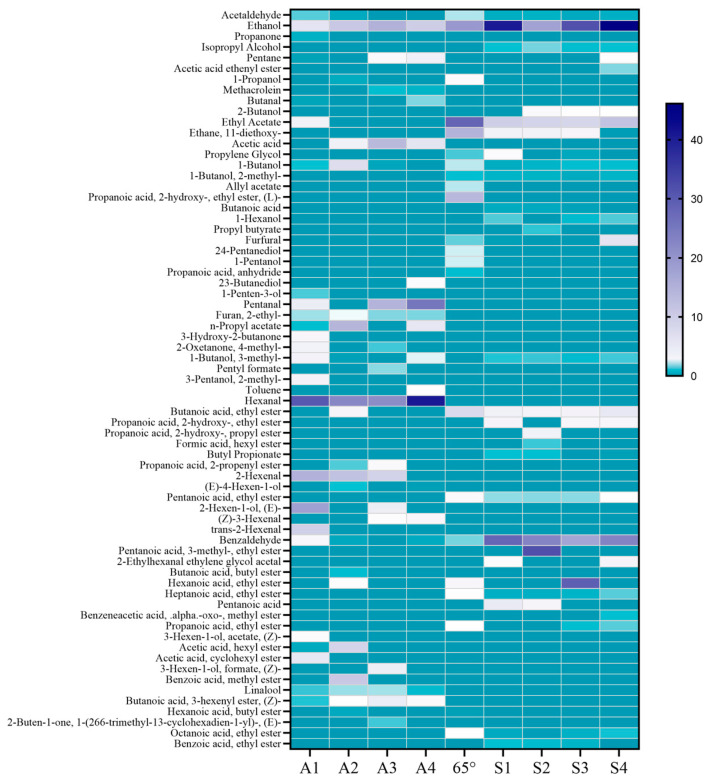
Heat map of aroma composition in Japanese apricot fruit (A1–A4), 65%vol base wine, and steeped mume wine made from 65%vol base wine (S1–S4).

From the 103 volatile components present in the fruit, the 65%vol base wine, and the steeped mume wine made from the 65%vol base wine, 69 representative substances with significant variations were selected to make a heat map, as shown in Figure 7. It is obvious that substances with 0–1% of aroma components are the majority of the graph, and that often components with more than 1% in the fruit are not as abundant within the macerated wines, and vice versa. Valeraldehyde, acetic acid, hexanal, 2-hexenal, (Z)-3-hexenal, and butanoic acid-3-hexenyl ester were high in the fruit at several periods but low in the steeped mume wine. Ethyl acetate, ethane, 1,1-diethoxy-, butanoic acid, ethyl ester, propanoic acid, 2-hydroxy-, ethyl ester, benzaldehyde, and pentanoic acid were substances that were present at levels greater than 1% within the steeped mume wines, but which were present at very low levels or even absent from the fruit. The 65%vol base wines were richer in aromatic components than 42%vol base wines, with more than 1 percent of ethanol, ethyl acetate, ethane, 1,1-diethoxy-, propanoic acid, 2-hydroxy-, ethyl ester, (L)-butanoic acid, ethyl ester, hexanoic acid, ethyl ester, and others. Ethanol was the only substance present in more than 10 percent of the fruit, the base wine, and the steeped mume wine(See annexed Tables S1 and S4 for more aroma components data).

### 2.5. Effect of Different Fruit Development Periods on the Nutrition Content of Fruit and Steeped Mume Wine

**Figure 8:**
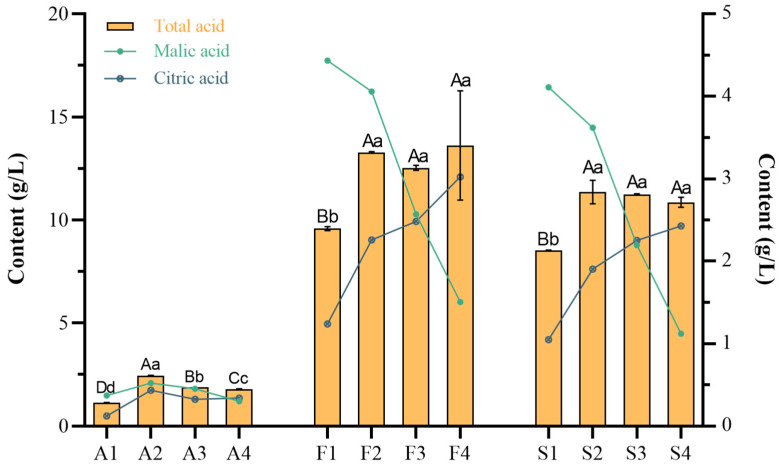
Content of organic acid in Japanese apricot fruit and steeped mume wine. Note: the amount of organic acid in Japanese apricot fruit (A1–A4), in steeped mume wine made from 42%vol base wine (F1–F4), and in steeped mume wine made from 65%vol base wine (S1–S4). Different small and capital letters indicate significant difference (*p* < 0.05) and extremely significant difference (*p* < 0.01) respectively.

Four (4) organic acids were detected within the fruit, in descending order of content: malic, citric, oxalic, and tartaric acids. Tartaric acid was not detected within the steeped mume wine. Figure 8 only shows the change trend in malic acid and organic acid content. As can be seen in Figure 8, the total amount of organic acids within the fruit was highest at 4.88 g/L in A2 and lowest at 1.14 g/L in A1, and the amount of citric acid was also highest at 1.73 g/L in A2 and lowest at 0.49 g/L in A1. It can be seen that the total amount of organic acids within the two steeped mume wines was low in the first period, and the amount was significantly higher in the second period. From then on, the total organic acid content did not change significantly. As can be seen in Figure 4, the content of citric acid in the steeped mume wines increased with fruit ripeness, while the content of malic and oxalic acids gradually decreased(See annexed Table S5 for more data).

**Figure 9:**
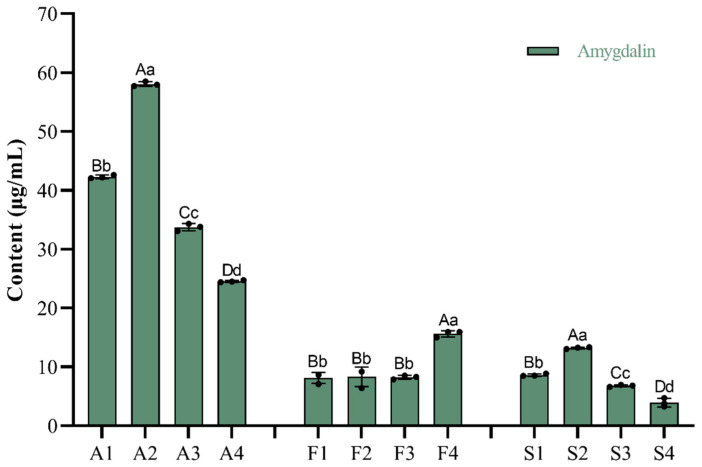
Content of amygdalin in Japanese apricot fruit and steeped mume wine. Note: content of amygdalin in Japanese apricot fruit (A1–A4), steeped mume wine made from 42%vol base wine (F1–F4), and steeped mume wine made from 65%vol base wine (S1–S4). Different small and capital letters indicate significant difference (*p* < 0.05) and extremely significant difference (*p* < 0.01) respectively.

As shown in Figure 9, the amygdalin content within the fruit at the A2 stage reached a peak value of 58.05 μg/mL. After that, with the increase in fruit maturity, the amygdalin content decreased, and at A4, it had the minimum value of 24.56 μg/mL. Within the steeped mume wine, the amygdalin content from F1 to F3 did not change significantly, and the content at F4 increased significantly to 20.97 μg/mL. The trend of S1 to S4 was more similar to that of A1 to A4, and S2 appeared to have the maximum value of 13.22 μg/mL (See annexed Table S6 for more data).

**Figure 10:**
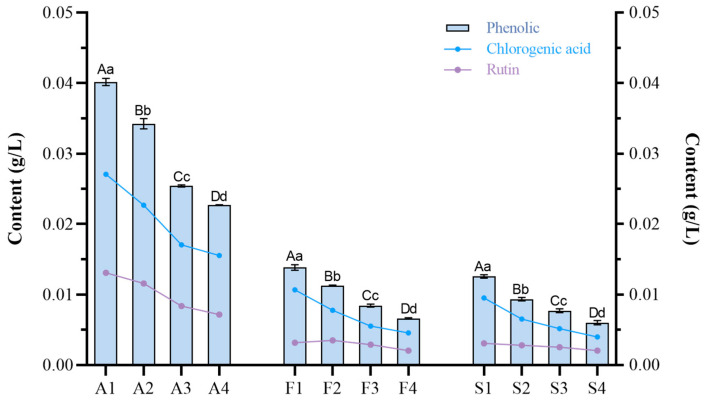
Content of phenolic in Japanese apricot fruit and steeped mume wine. Note: content of phenolic in Japanese apricot fruit (A1–A4), steeped mume wine made from 42%vol base wine (F1–F4), and steeped mume wine made from 65%vol base wine (S1–S4). Different small and capital letters indicate significant difference (*p* < 0.05) and extremely significant difference (*p* < 0.01) respectively.

Chlorogenic acid and rutin belong to polyphenols. As can be seen in Figure 10, the content of phenolics in the fruit and steeped mume wine decreased significantly at all times as the fruit matured. At the A1 period, the chlorogenic acid content was 0.0271 g/L, and the content of rutin was 0.0128 g/L, with chlorogenic acid being almost twice as high as rutin. By A4, the chlorogenic acid content was only 0.0155 g/L, and the rutin content was only 0.0070 g/L. Chlorogenic acid content decreased more rapidly than rutin, by 0.0611 g/L and 0.0610 g/L from F1 to F4 and S1 to S4, respectively, while rutin decreased by 0.0113 g/L and 0.0098 g/L, respectively (See annexed Table S7 for more data).

### 2.6. Effect of Microwave Aging on the Aroma Composition of Steeped Mume Wine

**Figure 11:**
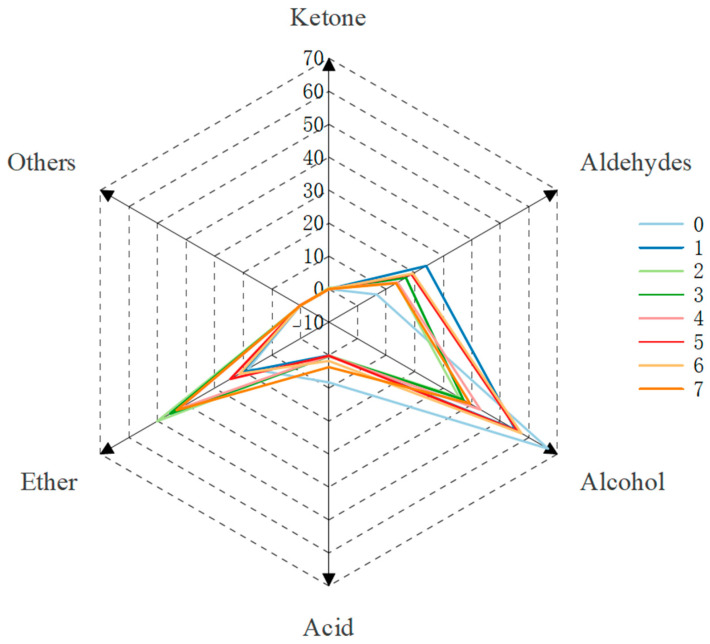
Radar chart of aroma composition after different aging treatments.

As shown in Figure 11, compared with the control group, microwave aging can significantly increase the contents of esters and aldehydes in fruit wines and reduce the contents of acids. Comparing groups 0, 1, 2, and 3, the low temperature of 40 °C can better promote the generation of aldehydes, and the total amount of esters and aldehydes is the most at 45 °C and 50 °C. Comparing groups 0, 2, 4, and 5, it can be seen that the treatment time has a great influence on the generation of esters: with the increase of the treatment time, the content of esters firstly increases and then decreases, and the content is the highest when the treatment time reaches 80 min. Comparing groups 0, 2, 6, and 7, the power conditions also had a great influence on the catalytic effect, with either too high or too low power affecting the generation of esters, and only when the power was 500 W, the acids in the fruit wines could be eliminated most effectively (Table 1).

**Figure 12:**
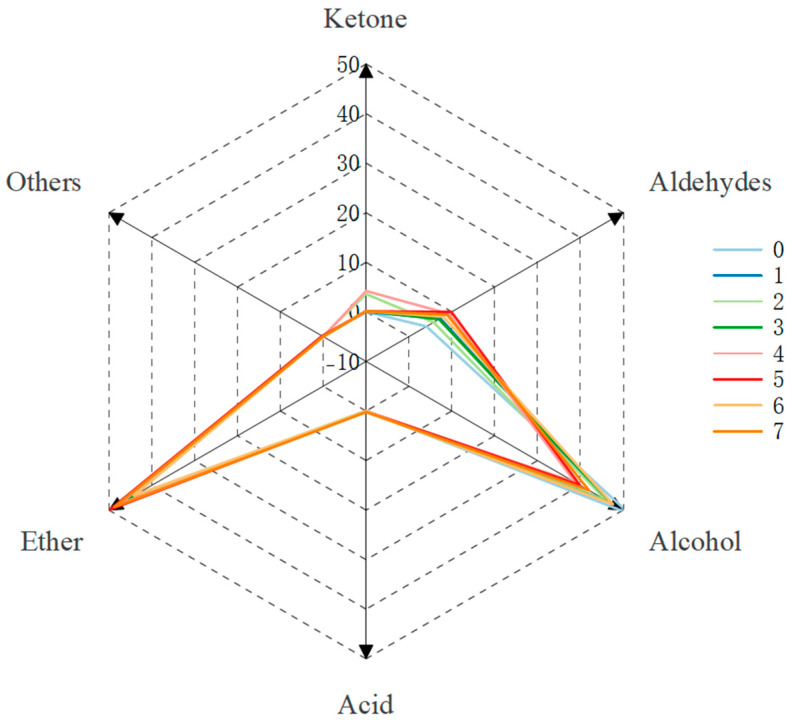
Radar chart of aroma composition after aging treatment for 4 months.

As can be seen in Figure 12, the groups of aroma components within the steeped mume wines changed dramatically again after 4 months of catalyzing treatment. The percentage of substances within each group became very close. The total percentage of esters ranged from 44 to 50 percent, the total percentage of aldehydes all decreased to less than 10 percent, the percentage of alcohols increased, and the rest of the substances were lower in content. Only Groups 2 and 4 had a higher percentage of acids, with total acid content percentages of 3.6% and 4.2%, respectively. Taken together, Group 5 had the highest total amount of esters and aldehydes, Group 6 had the lowest number of esters, and the control group had the lowest number of aldehydes (Table 2).

**Figure 13:**
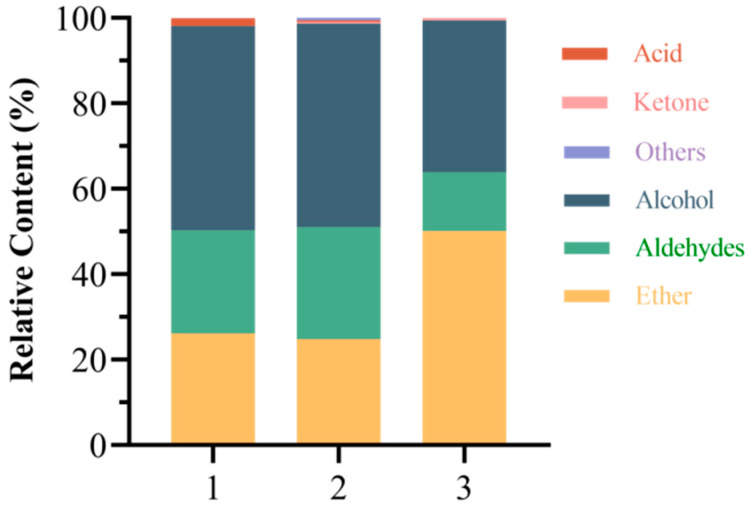
Comparison of the relative contents of aroma components between new wine and steeped mume wine after different aging methods. Note: 1, 2, and 3 indicate the wine of the year, the wine naturally aged for 10 years, and the wine after microwave aging, respectively.

**Figure 14:**
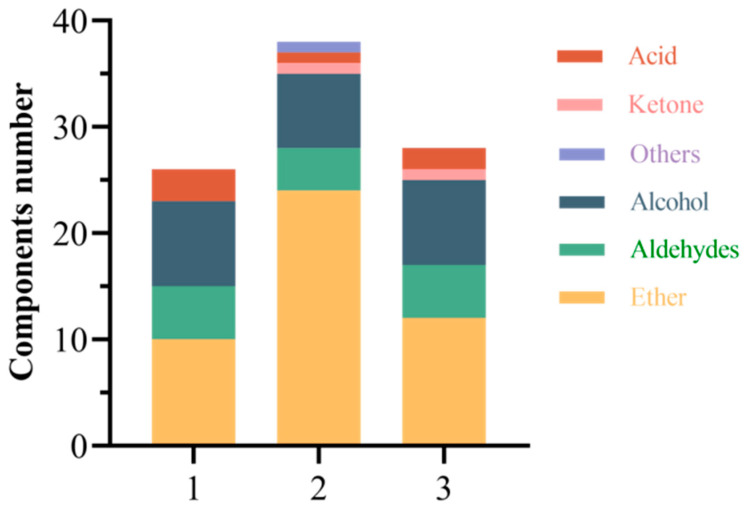
Comparison of the number of aroma components between new wine and steeped mume wine after different aging methods. Note: 1, 2, and 3 indicate the wine of the year, the wine naturally aged for 10 years, and the wine after microwave aging, respectively.

Three types of steeped mume wine with 65%vol base wine, namely, new wine of the year, base wine naturally aged for 10 years and microwave-activated new wine of the year, were compared to test the relative percentages and numbers of types of aroma constituents, and the results are shown in Figure 13. Compared with that of new wine of the year, the relative percentages of aroma constituents of the naturally aged wine of the year aged for 10 years did not change much, and the contents of esters, aldehydes, and alcohols hardly changed. Compared with the new wine of the same year, the relative percentages of aroma components in the wine aged naturally for 10 years did not change much; the contents of esters, aldehydes, and alcohols hardly changed; and the acids decreased slightly. After microwave treatment of new wines of the same year, the content of esters increased significantly, and the content of aldehydes, alcohols, and acids decreased accordingly. From the comparison of the number of aroma components in Figure 14, it can be concluded that the components of macerated wines underwent chemical reactions during the natural resting process, with increased esters and ketones and decreased aldehydes and acids in fruit wines. However, the number of esters did not change in the wines after microwave aging, and the number of acids decreased, with a small number of ketones added.

## 3. Discussion

Japanese apricot fruits, distinguished by their high acidity and low sugar levels, are naturally suited for transformation into processed products. Notably, their distinctive fruit aroma and nutrients can be transferred in the derived product—fruit wine when making macerated wine. A key objective of this study is to ascertain whether the peak period of fruit aroma coincides with the optimal time for producing fruit wine with the finest aroma. This information is crucial for pinpointing the ideal harvest time for fruit wine production. Concurrently, the nutritional profile of Japanese apricot fruit at various stages of development serves as supplementary data to identify the optimal harvest time. Additionally, this study delves into the investigation of innovative artificial aging methods for fruit wine. These methods are poised to expedite the production process, minimize costs, and enhance the overall quality of the wine, thereby offering significant advantages in the fruit wine industry.

The maturation process of Japanese apricot fruit is marked by a transformation in its aroma profile, shifting from green and aldehydic notes in immature stages to a predominance of fruity and floral scents in mature fruits [14,15]. Notably, the A2 phase of ‘Nannong Longxia’ fruit development is identified as the apex of aroma richness, characterized by high levels of esters such as propyl acetate and hexyl acetate, which impart a soft, fruity aroma reminiscent of holly oil. Simultaneously, the elevated levels of nutrients in A2 further corroborate the notion that this period is indeed the optimal time for harvesting. Concurrently, aldehydes like hexanal and 2-hexenal contribute a pungent and green leafy note, respectively. Nonetheless, during the A3 phase and beyond, there was a notable decline in ester content coupled with a rise in the concentration of pungent odors, adversely impacting the fruit’s aroma profile. 

The alcohol content in the steeped mume wine was reduced compared to that of the base wine due to the addition of rock sugar, which increased the quality of the wine and reduced its alcohol content. Consequently, the base wine originally at 42%vol has seen its alcohol content decrease to 20%vol, while a wine initially at 65%vol has been reduced to 30%vol. In steeped mume wine, the aroma composition is influenced by the ripeness of the fruit and the degree of the base wine. The 42%vol base wine provides a more stable aroma profile, while the 65%vol base wine aligns more closely with the fruit’s aroma, suggesting a strategic selection based on the desired outcome. The three most abundant substances in the wine—ethanol, benzaldehyde, and ethyl acetate—account for approximately 78.3% of the total volatile components, indicating their critical role in the wine’s aroma. The presence of benzaldehyde, which is produced in large quantities during maceration, is particularly significant, as it is a characteristic aroma constituent of the wine [16]. The sensory evaluation of steeped mume wine showed that people tended to give higher ratings to S2, as its aroma was stronger and accompanied by fruity notes. This is consistent with our GC-MS detection results: when using fruits with a better aroma to make fruit wine, the aroma of the resulting fruit wine was also more favored by people.

Amygdalin, a key component in traditional Chinese medicine, is utilized for treating cough and asthma, as an auxiliary cancer therapy, and for combating tissue fibrosis. While amygdalin itself is non-toxic, its metabolite, hydrogen cyanide, is highly poisonous. Hydrocyanic acid interferes with cellular respiration by inhibiting the activity of cytochrome oxidase in mitochondria, potentially leading to tissue asphyxiation and, in severe cases, death. The minimum lethal dose of hydrogen cyanide for humans is 0.4–1.0 mg/kg, meaning that 24 mg could be poisonous and 60 mg potentially fatal for a 60 kg adult. This study found the highest amygdalin level in steeped mume wine to be 21 μg/mL, equating to potential hydrocyanic acid poisoning in adults consuming over 1.14 L of the wine daily. These findings are preliminary and warrant further investigation.

Microwave aging technology is shown to enhance the sensory quality of fruit wine by disrupting molecular clusters, reducing ethanol’s pungency, and accelerating esterification reactions, thereby enriching the wine’s aroma [17,18]. The technology’s thermal and non-thermal effects promote chemical equilibrium and reactions, leading to faster wine maturation [19]. The potential for ‘reversion’, or a return to harsh flavors, post-aging is discussed, highlighting the need for continued research into cost-effective, versatile composite aging methods to address challenges in wine production [20]. Finally, this study examines the impact of aging methods on wine aroma, noting differences between natural and artificial aging. While natural aging preserves the proportion of aroma compounds, artificial aging rapidly increases ester content. This study suggests that composite aging techniques, combining methods like high-pressure and oak barrel aging, will likely gain more attention in the future due to their potential to enhance wine quality and shorten aging times [21,22,23].

## 4. Materials and Methods

### 4.1. Experimental Materials

The materials employed in this study were sourced from the National Field of Waxberry and Prunus mume at Nanjing Agricultural University, Nanjing, Jiangsu Province. Fruits were collected after the end of the fruit’s hard-core period. The variety of Japanese apricot fruit was Nannonglongxia. It is a selected variety for both ornamental and fruit-bearing purposes. It was developed through hybridization, with Longyan serving as the paternal parent and Daqianti as the maternal parent. This variety originated from Jiangsu, China, a region characterized by a subtropical monsoon climate. The area experiences abundant rainfall, with an annual precipitation of 1200 mm, and an average annual temperature of 15.4 °C. Nannonglongxia was planted as a grafted seedling, using Prunus mume as the rootstock. By 2024, the trees had reached an age of 10 years. Throughout the growth process, earthworm manure organic fertilizer and microbial fertilizer were applied to promote healthy development. In terms of pest control, sex pheromone disorientation filaments were utilized to interfere with the damage caused by heartworms to new shoots. Imidacloprid was applied to effectively control aphids, while mancozeb was used to manage scab disease. 

A sample was taken every week until the fruit reached maturity (Table 3). Basic wines (BWs), which are 42%vol and 65%vol (the volume of ethanol in wine at 20 °C), were used to prepare the mume wine steeped with the 4 samples below, named F1–F4 and S1–S4, respectively, as shown in Table 3. Base wine is obtained by steaming sorghum that has been fully soaked in water; mixing it with distiller’s yeast; and then subjecting it to saccharification, fermentation, and distillation. For each sample, we used 2 L (liter) of base wine and 1 kg (kilogram) of fruit. The method for crafting steeped mume wine involved macerating the whole Japanese apricot fruit and rock sugar in a base wine, with a specific mass ratio of 4:5:10 for sugar, fruit, and wine, respectively. Begin by washing the fruits, allowing them to air dry, and removing their stems. Arrange the Japanese apricot fruit and rock sugar in layers within a clear glass container, then pour in the base wine until it reaches the rim of the bottle. Securely seal the bottle and tightly wrap the mouth with a layer of transparent film to ensure an airtight closure. Store the bottle at a temperature of 25 °C for a period of three months before conducting any quality assessments. During the maceration, pictures were taken of samples to record the wine’s color changes at specific times. These were 1 day, 1 week, 2 weeks, 3 weeks, 4 weeks, 8 weeks, 12 weeks, and 14 weeks of maceration. Both the fruit and wine samples were subjected to biological duplication.

### 4.2. Qualitative Analysis by HS-SPME-GC-MS

Headspace Solid-Phase Micro Extraction/Gas Chromatography–Mass Spectrometry (HS-SPME-GC-MS) can quantitatively analyze the volatile components in the fruit and wine, and the volatile compounds were qualitatively identified by the NIST 2017 database. The fruit was powdered with liquid nitrogen and weighed to 3.0 g; then, 3 mL of saturated sodium chloride solution was added, shaken well, and sealed for analysis. Next, 5 mL of infused mume wine was accurately measured into a 20 mL tube; then, 1.5 g of NaCl was added, shaken well, and extracted with SPME fiber (75 μm CAR/PDMS) prior to testing. The experimental instrument was the gas chromatography–triple quadrupole mass spectrometer (TSQ 9000/ Trace 1310, Thermo Fisher Scientific-CN, Shanghai, China), and the chromatographic column was a TG-5MS capillary column (30 m × 0.25 mm × 0.25 μm, Thermo Fisher Scientific). The carrier gas was He (Helium) (99.999%), with a flow rate of 1.0 mL/min and a split ratio of 4:1. The injection port temperature was 250 °C, and the temperature program was as follows: the column temperature was initially maintained at 50 °C for 6 min, then increased at a rate of 3 °C/min to 120 °C for 2 min, and then increased at a rate of 10 °C/min to 260 °C for 2 min. The mass spectrometry conditions were as follows: the GC-MS interface temperature was 270 °C; the ion source temperature was 280 °C, using electron ionization (EI) with an electron energy of 70 eV; the scan mass range was 33–350 *m*/*z*.

### 4.3. Quantitative Analysis by UPLC 

#### 4.3.1. The Injection Procedures of UPLC

The contents of nutrient composition can be accurately measured by Ultra-Performance Liquid Chromatography (UPLC). The detector of ultra-high-performance liquid chromatography is the photo-diode array (PDA).The solutions were filtered through a 0.22 μm membrane. Subsequently, the solutions were injected under the chromatographic conditions of the Waters ACQUITY UPLC HSS T3 column (2.1 mm × 100 mm, 1.8 μm; Shanghai, China) to generate standard curves for each component. The individual content of oxalic acid, tartaric acid, malic acid, citric acid, amygdalin, chlorogenic acid, and rutin was determined separately. The injection procedures for the different components when performing UPLC assays were as follows. For the organic acid measurement, the mobile phase was 20 mM KH_2_PO_4_ (with 1% methanol), and the flow rate was set at 0.25 mL/min. The column temperature was maintained at 30 °C, with 5 min for the analysis time, and the injection volume was 2 µL. For amygdalin measurement, the mobile phase consisted of 75% water and 25% methanol, and the flow rate was set at 0.2 mL/min. The column temperature was maintained at 25 °C, 8 min for the analysis time, and the injection volume was 2 µL. For the measurement of phenolics compounds, the mobile phase consisted of 0.1% acetic acid and acetonitrile, and the flow rate was set at 0.35 mL/min. The column temperature was maintained at 30 °C, analysis time was 5 min, and the injection volume was 2 µL.

#### 4.3.2. Preparation of Organic Acid Samples and Calibration Curves

Organic acids were extracted from the fruit by initially pulverizing 0.5 g of the fruit sample into a fine powder using liquid nitrogen. Subsequently, 1.5 mL of 80% ethanol solution was added, and the mixture was placed in a water bath at 80 °C for 30 min. The solution was then centrifuged at 20,913× *g* for 10 min, and the supernatant was collected. The extraction process was repeated once, and the combined supernatants from both extractions were vacuum freeze-dried. After freeze-drying, 10 mL of ultrapure water was added to dissolve the extract, which was mixed thoroughly and filtered through a 0.22 μm microfilm for future use. For the calibration curves, 0.1 g of oxalic acid, tartaric acid, malic acid, and citric acid was accurately weighed and dissolved in ultrapure water to prepare a mixed standard solution with a concentration of 1 mg/mL. The standard solution was then diluted with ultrapure water to obtain gradient reference standard solutions with concentrations of 1 mg/mL, 0.5 mg/mL, 0.1 mg/mL, 0.05 mg/mL, and 0.001 mg/mL. Calibration curve equations please see Table S8 in Supplementary Materials.

#### 4.3.3. Preparation of Amygdalin Samples and Calibration Curves

Amygdalin acids were extracted from the fruit by initially pulverizing 0.5 g of the fruit sample into a fine powder using liquid nitrogen. Then, 3 mL of 60% ethanol solution was added, and the mixture was placed in a 60 °C water bath for 30 min, followed by sonication in an ultrasonic cleaner for 10 min. The solution was then centrifuged, and the supernatant was collected. The extraction process was repeated once, and the combined supernatants from both extractions were vacuum freeze-dried. After freeze-drying, 8 mL of methanol was added to dissolve the extract, which was mixed thoroughly and filtered through a 0.22 μm microfilm for future use. For the preparation of standard solutions, 40 mg of amygdalin standard substance was accurately weighed and dissolved in methanol to prepare a standard solution with a concentration of 400 μg/mL. This standard solution was further diluted with methanol to obtain gradient reference standard solutions with concentrations of 80 μg/mL, 40 μg/mL, 20 μg/mL, 10 μg/mL, and 5 μg/mL. Calibration curve equations please see Table S8 in Supplementary Materials.

#### 4.3.4. Preparation of Phenolics Samples and Calibration Curves

Phenolics acids were extracted from the fruit by initially pulverizing 6 g of the fruit sample into a fine powder using liquid nitrogen. A 60% ethanol solution was added at a ratio of 1:7 (42 mL), and the mixture was extracted at 24 °C for 24 h. The extract was then subjected to ultrasonication for 30 min and centrifuged at 1966× *g* for 10 min, with the supernatant collected. The process of ultrasonication and centrifugation was repeated once, and the two supernatants were combined and filtered through a 0.22 μm microfilm for future use. For the preparation of the standard solutions, 2 mg each of chlorogenic acid and rutin standard substances were accurately weighed and dissolved in 60% ethanol solution to prepare a mixed standard solution with a concentration of 0.2 mg/mL. This mixed standard solution was further diluted with 60% ethanol solution to obtain gradient reference standard solutions with concentrations of 0.02 mg/mL, 0.01 mg/mL, 0.005 mg/mL, 0.0025 mg/mL, and 0.001 mg/mL. Calibration curve equations please see Table S8 in Supplementary Materials.

#### 4.3.5. Preparation of Steeped Mume Wine

The steps for preparing a sample of steeped mume wine are as follows: First, 10 mL of steeped mume wine was pipetted into a 50 mL centrifuge tube and placed into a freeze dryer for 3–4 days until the liquid was completely evaporated. An equal volume of ultrapure water, methanol, or 60% ethanol solution was then added to the dried sample to measure the contents of organic acids, amygdalin, and phenols, respectively. The mixture was subjected to ultrasonic treatment in an ultrasonic cleaning machine for 20 min to ensure complete dissolution. The solution was then centrifuged at 1966× *g* for 20 min, and the supernatant was collected. Finally, the supernatant was filtered through a 0.22 μm pore size filter to obtain the sample for testing. The calibration curves used were the same as described previously.

### 4.4. Aging Reaction of Steeped Mume Wine

Referring to the method of Jiang Qian [24], a microwave synthesis reactor was used to age the steeped mume wine. Temperature, power, and time were considered as the three main factors affecting the effectiveness. A total of 50 mL of fruit wine was added to a three-necked flask, stirring magnets were added, and the flask was placed smoothly on a gasket in the center of the reactor. A glass tee tube was inserted inside the reactor to connect with the mouth in the middle of the three-necked flask, the left port of the tee tube was connected to the spherical condenser tube, and the middle and right ports were plugged with rubber stoppers. The probe of the sensor inside the reactor was connected to the left port of the three-necked flask through the silicone plug to monitor the temperature of the fruit wine during the reaction. The mouth of the right flask was plugged with a rubber stopper. The treatment conditions and group numbers are shown below (Table 4). Each process was repeated 3 times.

### 4.5. Data Statistics and Analysis 

SPSS Statistics (Version 27.0, International Business Machines Corporation, Armonk, NY, USA) for analysis of variance and the significance test [LSD (Least Significant Difference), *p* < 0.05] were used. GraphPad prism 8 (GraphPad Software 8.0.2) was used for further analysis and plotting. The sample’s relative percentage of volatile components was calculated using the internal standard method and peak area. 3-octanol was added to the sample as an internal standard substance. The peak area and concentration of 3-octanol can be used to calculate the content of each aroma component, thus calculating the percentage of each component according to the total amount. The content of nutrients in the samples was calculated using the internal standard method.

## 5. Conclusions

Our study reveals that the most opportune moment to harvest fruit for crafting steeped mume wine is 81 days post-flowering, when the fruit’s aromatic compounds, especially esters, are most abundant, and its nutritional profile is optimal. Analysis of the wine’s composition after microwave aging suggests that the ideal aging parameters are a temperature of 50 °C for 80 min at a power level of 500 watts. These insights lay a theoretical foundation for identifying the peak aromatic period of macerated Japanese apricot fruits and steeped mume wine, pinpointing the best harvest time and evaluating novel aging techniques. The interaction and transfer of aromatic and nutritional compounds from Japanese apricot fruit to mume wine during the infusion process is a fascinating area that merits deeper investigation. Furthermore, the conclusions drawn regarding steeped mume wine necessitate substantial empirical data for validation.

## Figures and Tables

**Table 1:** Percentage of aroma components in steeped mume wine after different aging treatments.

Component Type	BP Area Relative Content (%)							
	0	1	2	3	4	5	6	7
Ether	18.15	20.47	50.16	45.58	42.57	24.53	21.33	43.36
Aldehydes	6.76	24.08	13.72	16.98	14.09	18.93	19.47	13.54
Alcohol	66.78	55.30	35.61	37.11	43.10	56.25	57.39	39.44
Ketone	/	/	0.38	/	/	/	/	/
Acid	8.30	0.09	0.13	0.34	0.24	0.17	1.81	3.65
Others	0.00	0.05	/	/	0.00	0.11	/	0.02

**Table 2:** Percentage of aroma components in steeped mume wine after aging treatment for 4 months.

Component Type	BP Area Relative Content (%)							
	0	1	2	3	4	5	6	7
Ether	44.34	46.37	44.45	45.33	47.56	49.78	44.17	48.72
Aldehydes	4.11	7.32	5.76	7.03	9.28	9.94	8.03	8.88
Alcohol	51.39	46.00	46.01	47.39	38.82	39.89	47.62	42.04
Ketone	0.01	0.02	3.61	0.19	4.22	0.11	0.13	0.14
Acid	0.02	0.04	0.03	0.04	0.12	0.07	0.05	0.22
Others	0.12	0.24	0.13	0.01	/	0.22	/	/

**Table 3:** Japanese apricot fruit and steeped mume wine materials.

Sample	Material	Sample	Material	Sample	Material
A1	74 DAF	F1	2 L 42%vol BW + 1 kg A1	S1	2 L 65%vol BW + 1 kg A1
A2	81 DAF	F2	2 L 42%vol BW + 1 kg A2	S2	2 L 65%vol BW + 1 kg A2
A3	88 DAF	F3	2 L 42%vol BW + 1 kg A3	S3	2 L 65%vol BW + 1 kg A3
A4	95 DAF	F4	2 L 42%vol BW + 1 kg A4	S4	2 L 65%vol BW + 1 kg A4

**Table 4:** The groups and treatment conditions.

Group Number	Temperature	Power (Watt)	Time
Control Group (0)	**/**	**/**	**/**
1	40 °C	500 W	80 min
2	45 °C	500 W	80 min
3	50 °C	500 W	80 min
4	45 °C	500 W	40 min
5	45 °C	500 W	60 min
6	45 °C	400 W	80 min
7	45 °C	600 W	80 min

## Data Availability

The original contributions presented in this study are included in the article and Supplementary Materials.

## Supplementary Materials

The following supporting information can be downloaded at: https://www.mdpi.com/article/10.3390/molecules30020392/s1, Table S1: Volatile component and BP area relative content in Japanese apricot fruit. Table S2: Volatile component and BP area relative content in steeped mume wine. Table S3: Volatile component and BP area relative content in steeped mume wine. Table S4: Aroma components and relative percentage contents of base wine. Table S5: Organic acid content of Japanese apricot fruit and steeped mume wine (g/L). Table S6: Organic acid content of Japanese apricot fruit and steeped mume wine (g/L). Table S7: Phenolic content of Japanese apricot fruit and steeped mume wine (g/L). Table S8: Calibration curve equations.
