# Supplementary material for: Effect of Different Harvest Time and Microwave Aging on Aroma Characteristics of Japanese Apricot Wine
Source: Molecules. 2025 Jan 18;30(2):392. doi: 10.3390/molecules30020392 (PMC11767645; doi:10.3390/molecules30020392)
Supplement: Supplementary file 1 [file molecules-30-00392-s001.zip › molecules-3392128-supplementary.pdf]

Annexed Table

Table S1 Volatile component and BP area relative content in Japanese apricot fruit

Number	Component Name	CAS	Formula	BP Area Relative Content（%）			
				A1	A2	A3	A4
1	Acetaldehyde	75-07-0	C2H4O	0.49	0.13	/	/
2	Ethanol	64-17-5	C2H6O	5.93	10.74	14.97	9.90
3	Propanone	67-64-1	C3H6O	0.21	/	/	/
4	Pentane	109-66-0	C5H12	0.06	/	2.04	3.68
5	1-Propanol	71-23-8	C3H8O	/	0.15	/	/
6	Methacrolein	78-85-3	C4H6O	/	/	0.35	0.24
7	Butanal	123-72-8	C4H8O	0.09	/	/	0.59
8	Ethyl Acetate	141-78-6	C4H8O2	3.42	/	/	/
9	Acetic acid	64-19-7	C2H4O2	/	3.60	13.56	5.68
10	1-Butanol	71-36-3	C4H10O	0.38	7.10	0.10	/
11	2,3-Butanediol	123513-85-9	C4H10O2	/	/	/	1.54
12	1-Penten-3-ol	616-25-1	C5H10O	0.47	/	/	/
13	Pentanal	110-62-3	C5H10O	4.26	/	14.53	25.24
14	Furan, 2-ethyl-	3208-16-0	C6H8O	0.68	0.95	0.59	0.58
15	n-Propyl acetate	109-60-4	C5H10O2	0.35	14.26	/	5.32
16	3-Hydroxy-2-butanone	513-86-0	C4H8O2	2.67	/	/	/
17	2-Oxetanone, 4-methyl-	3068-88-0	C4H6O2	3.13	/	0.45	/
18	1-Butanol, 3-methyl-	123-51-3	C5H12O	3.16	0.05	/	0.89
19	Pentyl formate	638-49-3	C7H5Cl3	/	/	0.62	/
20	3-Pentanol, 2-methyl-	565-67-3	C6H14O	3.18	/	/	/
21	Toluene	108-88-3	C7H8	/	/	/	1.08
22	Hexanal	66-25-1	C6H12O	30.37	22.25	21.25	40.45
23	Butanoic acid, ethyl ester	105-54-4	C6H12O2	/	2.92	/	/
24	Propanoic acid, 2-propenyl ester	2408-20-0	C6H10O2	/	0.48	1.95	/
25	2-Hexenal	505-57-7	C6H10O	15.14	12.1	9.05	/
26	(E)-4-Hexen-1-ol	928-92-7	C6H12O	/	0.38	/	0.04
27	P-Xylene	106-42-3	C8H10	0.06	/	/	0.04
28	Ethylbenzene	100-41-4	C8H10	/	0.13	/	/
29	2-Hexen-1-ol, (E)-	928-95-0	C6H12O	17.90	/	4.28	/
30	(Z)-3-Hexenal	6789-80-6	C6H10O	/	/	1.48	2.36
31	trans-2-Hexenal	6728-26-3	C6H10O	9.60	/	/	/
32	Benzaldehyde	100-52-7	C7H6O	2.36	0.12	/	0.14
33	1,3-Cyclohexadiene, 1-methyl-4-(1-methylethyl)-	10482-56-1	C10H18O	/	/	0.06	/
34	Butanoic acid, butyl ester	109-21-7	C8H16O2	/	0.34	/	/
35	Hexanoic acid, ethyl	123-66-0	C8H16O2	/	1.52	/	/

	ester						
36	3-Hexen-1-ol, acetate, (Z)-	3681-71-8	C8H14O2	1.54	/	/	/
37	Acetic acid, hexyl ester	142-92-7	C8H16O2	0.15	8.93	/	/
38	Acetic acid, cyclohexyl ester	622-45-7	C8H14O2	5.17	/	/	/
39	1-Pentanol, 4-methyl-	626-89-1	C6H14O	0.07	/	/	/
40	3-Hexen-1-ol, formate, (Z)-	33467-73-1	C7H12O2	/	/	4.03	/
41	Benzoic acid, methyl ester	93-58-3	C8H8O2	/	10.88	/	/
42	Linalool	78-70-6	C10H18O	0.43	0.67	0.70	0.32
43	Butanoic acid, 3-hexenyl ester, (Z)-	16491-36-4	C10H18O2	0.41	1.15	4.42	1.89
44	alpha.-Terpineol	98-55-5	C10H18O	/	0.07	0.09	/
45	Hexanoic acid, butyl ester	626-82-4	C10H20O2	/	0.13	/	/
46	2-Furanmethanol, tetrahydro-, acetate	637-64-9	C7H12O3	0.18	/	/	/
47	1-Oxaspiro [4.5]dec-6-ene, 2,6,10,10-tetramethyl-	36431-72-8	C13H22O	/	0.04	/	0.03
48	2-Butanone, 4-(2,6,6-trimethyl-1-cyclohexen-1-yl)-	17283-81-7	C13H22O	/	0.05	/	/
49	2-Buten-1-one, 1-(2,6,6-trimethyl-1,3-cyclohexadien-1-yl)-, (E)-	23726-93-4	C13H18O	/	/	0.45	/
50	2(4H)-Benzofuranone, 5,6,7,7a-tetrahydro-4,4,7a-trimethyl-	15356-74-8	C11H16O2	/	0.02	0.02	/

Table S2 Volatile component and BP area relative content in steeped mume wine made by 42%vol

Number	Component Name	CAS	Formula	BP Area Relative Content（%）			
				F1	F2	F3	F4
1	Acetaldehyde	75-07-0	C2H4O	0.06	/	/	0.03
2	Ethanol	64-17-5	C2H6O	35.24	38.31	29.24	48.10
3	Ethyl Acetate	141-78-6	C4H8O2	16.02	11.89	16.36	11.18
4	Ethane, 1,1-diethoxy-	105-57-7	C6H14O2	/	0.14	0.14	0.05
5	Butanoic acid, ethyl ester	105-54-4	C6H12O2	/	/	/	0.02
6	Propanoic acid, 2-hydroxy-, ethyl ester	97-64-3	C5H10O3	4.83	/	/	/
7	Propanoic acid, 2-hydroxy-, propyl ester	616-09-1	C6H12O3	/	/	/	0.31
8	Propane, 2,2-dimethoxy-	77-76-9	C5H12O2	/	/	0.01	/
9	2-Hydroxy acetophenone	582-24-1	C8H8O2	0.07	/	/	/
10	Benzaldehyde	100-52-7	C7H6O	38.81	40.24	43.40	27.36
11	Pentanoic acid, 3-methyl-, ethyl ester	5870-68-8	C8H16O2	/	/	8.72	/
12	2-Ethylhexanal ethylene glycol acetal	/	C10H20O2	/	0.01	1.09	/
13	Benzyl alcohol	100-51-6	C7H8O	/	/	0.2	/
14	Propargyl alcohol	107-19-7	C3H4O	0.01		0.01	
15	Methyl formate	107-31-3	C2H4O2	/	4.24	/	/
16	Propene	115-07-1	C3H6	/	0.38	/	/
17	Hexanoic acid, ethyl ester	123-66-0	C8H16O2	4.95	0.39	0.5	12.62
18	Hexanoic acid, butyl ester	626-82-4	C10H20O2	0.00	/	/	/
19	Octanoic acid, ethyl ester	106-32-1	C10H20O2	/	0.03	/	/
20	Benzoic acid, ethyl ester	93-89-0	C9H10O2	/	0.16	0.31	0.29
21	Acetic acid, 1,1-dimethylethyl ester	540-88-5	C6H12O2	/	/	/	0.01
22	Benzaldehyde diethylacetal	774-48-1	C11H16O2	0.03	0.03	0.03	0.02

Table S3 Volatile component and BP area relative content in steeped mume wine made by 65%vol

Number	Component Name	CAS	Formula	BP Area Relative Content（%）			
				S1	S2	S3	S4
1	Acetaldehyde	75-07-0	C2H4O	0.18	0.23	0.14	0.19
2	Ethanol	64-17-5	C2H6O	40.28	17.50	31.43	46.11
3	Isopropyl Alcohol	67-63-0	C3H8O	0.38	0.56	0.34	0.38
4	Acetic acid ethenyl ester	108-05-4	C4H6O2	/	/	/	0.59
5	Propylene Glycol	57-55-6	C3H8O2	1.69	/	0.12	/
6	2-Butanol	78-92-2	C4H10O	/	1.89	1.32	1.02
7	Ethyl Acetate	141-78-6	C4H8O2	9.68	8.80	8.32	11.95
8	1-Butanol	71-36-3	C4H10O	0.16	0.25	0.34	0.35
9	2-Pentanol	6032-29-7	C5H12O	0.06	0.05	0.05	0.06
10	3-Pentanol	584-02-1	C5H12O	0.04	/	/	/
11	Propanoic acid, ethyl ester	105-37-3	C5H10O2	/	/	0.34	0.49
12	Butyl Propionate	20487-40-5	C7H14O2	0.37	0.36	/	0.04
13	Ethane, 1,1-diethoxy-	105-57-7	C6H14O2	3.35	3.44	2.88	0.02
14	1-Butanol, 3-methyl-	123-51-3	C5H12O	0.41	0.43	0.32	0.45
15	1-Butanol, 2-methyl-	137-32-6	C5H12O	0.24	0.23	0.15	0.24
16	Propanoic acid, 2-methyl-, anhydride	97-72-3	C8H14O3	/	/	0.02	0.01
17	Butanoic acid	107-92-6	C4H8O2	0.15	0.13	0.07	/
18	Paraldehyde	123-63-7	C6H12O3	/	/	/	0.01
19	Butanoic acid, ethyl ester	105-54-4	C6H12O2	3.49	2.95	3.20	5.14
20	Furfural	98-01-1	C5H4O2	0.04	/	/	006
21	1-Hexanol	111-27-3	C6H14O	0.48	/	0.33	0.48
22	Propyl butyrate	638-11-9	C7H14O2	/	0.42	/	/
23	Propanoic acid, 2-hydroxy-, ethyl ester	97-64-3	C5H10O3	2.99	/	2.74	2.63
24	Butanoic acid, 2-methyl-, ethyl ester	7452-79-1	C7H14O2	/	/	0.01	0.01
25	Propanoic acid, 2-methyl-, ethyl ester	97-62-1	C6H12O2	/	/	/	0.02
26	Butanoic acid, 3-methyl-, ethyl ester	108-64-5	C7H14O2	/	/	0.03	/
27	1-Pentanol, 4-methyl-	626-89-1	C6H14O	/	/	0.05	/
28	Propanoic acid, 2-hydroxy-, propyl ester	616-09-1	C6H12O3	/	3.41	/	/
29	Formic acid, hexyl ester	629-33-4	/	/	0.44	/	/
30	Pentanoic acid, ethyl ester	539-82-2	C7H14O2	0.64	0.61	0.62	1.09
31	Butane,	3842-3-3	C9H20O2	/	/	0.1	/

32	1,1-diethoxy-3-methyl- Benzaldehyde	100-52-7	C7H6O	28.21	22.81	17.14	23.02
33	Chloromethyl chloroacetate	6135-23-5	C3H4Cl2O 2	/	0.01	/	/
34	Pentanoic acid, 3-methyl-, ethyl ester	5870-68-8	C8H16O2	/	31.73	/	/
35	2-Ethylhexanal ethylene glycol acetal	/	C10H20O2	1.56	/	/	3.10
36	Pentane	109-66-0	/	/	/	/	1.24
37	Benzyl alcohol	100-51-6	C7H8O	/	0.11	0.09	0.01
38	4-Heptanol, 2,6-dimethyl-	108-82-7	C9H20O	/	0.02	0.03	/
39	1,2,3-Propanetriol, 1-acetate	106-61-6	C5H10O4	/	/	0.01	/
40	Heptanoic acid, ethyl ester	106-30-9	C9H18O2	0.20	0.19	0.25	0.49
41	Benzeneacetic acid, .alpha.-oxo-, methyl ester	15206-55-0	C9H8O3	/	/	/	0.36
42	Benzene, 1,2,3,5-tetramethyl-	527-53-7	C10H14	0.03	0.02	/	/
43	Hexanoic acid, ethyl ester	123-66-0	C8H16O2	/	/	29.15	/
44	Pentanoic acid	109-52-4	C5H10O2	4.83	2.88	0.02	/
45	Butanedioic acid, diethyl ester	123-25-1	C8H14O4	/	/	0.01	/
46	Hexanoic acid, butyl ester	626-82-4	C10H20O2	/	/	0.01	0.01
47	Octanoic acid, ethyl ester	106-32-1	C10H20O2	0.14	0.03	0.21	0.39
48	Benzoic acid, ethyl ester	93-89-0	C9H10O2	0.36	0.33	0.25	/
49	Benzaldehyde diethylacetal	774-48-1	C11H16O2	0.04	0.03	0.03	0.04
50	Butanedioic acid, hydroxy-, diethyl ester	7554-12-3	C8H14O5	0.02	/	0.02	0.02

Table S4 Aroma components and relative percentage contents of base wine

Number	Component Name	CAS	Formula	BP Area Relative Content（%）	
				42°Base Wine	65°Base Wine
1	Acetaldehyde	75-07-0	C2H4O	/	0.73
2	Ethanol	64-17-5	C2H6O	74.11	19.39
3	1-Propanol	71-23-8	C3H8O	/	1.73
4	Acetic acid ethenyl ester	108-05-4	C4H6O2	0.03	/
5	2-Propanol, 2-methyl-	75-65-0	C4H10O	/	0.08
6	Ethyl Acetate	141-78-6	C4H8O2	20.85	28.41
7	Allyl acetate	591-87-7	C5H8O2	/	0.76
8	1-Butanol	71-36-3	C4H10O	/	0.77
9	Carbonic acid, ethyl-, methyl ester	623-53-0	C4H8O3	/	0.05
10	Propanoic acid, ethyl ester	105-37-3	C5H10O2	/	1.00
11	Ethane, 1,1-diethoxy-	105-57-7	C6H14O2	0.10	14.71
12	1-Pentanol	71-41-0	C4H10O	/	0.83
13	1-Butanol, 2-methyl-	137-32-6	C5H12O	/	0.36
14	Propylene Glycol	57-55-6	C3H8O2	/	0.46
15	Butanoic acid, ethyl ester	105-54-4	C6H12O2	/	7.71
16	Propanoic acid, 2-hydroxy-, ethyl ester, (L)-	687-47-8	C5H10O3	2.62	13.67
17	2,4-Pentanediol	625-69-4	C5H12O2	/	0.83
18	Furfural	98-01-1	C5H4O2	/	0.52
19	Butanoic acid, 3-methyl-, ethyl ester	108-64-5	C7H14O2	/	0.05
20	ethylbenzene	100-41-4	C8H10	0.01	/
21	Pentanoic acid, ethyl ester	539-82-2	C7H14O2	0.47	1.93
22	3-Furaldehyde	498-60-2	C5H4O2	/	0.02
23	Benzaldehyde	100-52-7	C7H6O	0.14	0.57
24	Propanoic acid, anhydride	123-62-6	C6H10O3	/	0.35
25	Hexanoic acid, ethyl ester	123-66-0	C8H16O2	/	2.69
26	Propanoic acid, 2-methyl-, 2-methylbutyl ester	2445-69-4	C9H18O2	/	0.01
27	Pyruvic acid, butyl ester	20279-44-1	C7H12O3	/	0.03
28	Heptanoic acid, ethyl ester	106-30-9	C9H18O2	0.43	1.06

29	Phenylethyl Alcohol	98-85-1	C8H10O	/	/
30	Hexanoic acid, butyl ester	626-82-4	C10H20O2	/	0.04
31	Propanoic acid, 2-methyl-, hexyl ester	2349-07-7	C10H20O2	/	0.01
32	Hexanoic acid, propyl ester	626-77-7	C9H18O2	/	0.02
33	Benzoic acid, ethyl ester	93-89-0	C9H10O2	0.43	/
34	Octanoic acid, ethyl ester	106-32-1	C10H20O2	0.46	1.10
35	Isopentyl hexanoate	2198-61-0	C11H22O2	0.05	0.10

Table S5 Organic acid content of Japanese apricot fruit and steeped mume wine(g/L)

Sample	Malic acid	Citric acid
A1	0.38±0.001Cc	0.49±0.007Dd
A2	0.52±0.007Aa	1.73±0.009Aa
A3	0.45±0.003Bb	1.30±0.003Cc
A4	0.30±0.003Dd	1.36±0.003Bb
F1	4.43±0.02Aa	4.95±0.04Cc
F2	4.06±0.03Bb	9.04±0.01Bb
F3	2.57±0.01Cc	9.93±0.07Bb
F4	1.50±0.18Dd	12.10±1.35Aa
S1	4.11±0.01Aa	4.19±0.00Dd
S2	3.62±0.16Bb	7.61±0.19Cc
S3	2.20±0.02Cc	9.01±0.04Bb
S4	1.12±0.01Dd	9.71±0.14Aa

Note: Differences in nutrient content ($p < 0.05$) and different uppercase letters indicate extremely significant differences in nutrient content ($p < 0.01$).

Table S6 Amygdalin content of Japanese apricot fruit and steeped mume wine (μg/mL)

Sample	Amygdalin	Sample	Amygdalin	Sample	Amygdalin
A1	42.30±0.18Bb	F1	8.15±0.54Cc	S1	8.63±0.12Cc
A2	58.05±0.23Aa	F2	8.33±0.95Cc	S2	13.22±0.09Aa
A3	33.75±0.36Cc	F3	8.25±0.20Cc	S3	6.80±0.09Dd
A4	24.56±0.10Dd	F4	15.63±0.31Bb	S4	3.93±0.43Ee

Note: Content is presented as mean ± standard deviation. Different lowercase letters indicate significant differences in nutrient content ($p < 0.05$), and different uppercase letters indicate extremely significant differences in nutrient content ($p < 0.01$).

Table S7 Phenolic content of Japanese apricot fruit and steeped mume wine(g/L)

Sample	Phenolic	Chlorogenic acid	Rutin
A1	0.0399±0.00Aa	0.0271±0.00Aa	0.0128±0.00Aa
A2	0.0344±0.00Bb	0.0227±0.00Bb	0.0117±0.00Bb
A3	0.0254±0.00Cc	0.0171±0.00Cc	0.0083±0.00Cc
A4	0.0225±0.00Dd	0.0155±0.00Dd	0.0070±0.00Dd
F1	0.0139±0.00Aa	0.0107±0.00Aa	0.0032±0.00Bb
F2	0.0112±0.00Bb	0.0077±0.00Bb	0.0035±0.00Aa
F3	0.0084±0.00Cc	0.0055±0.00Cc	0.0029±0.00Bb
F4	0.0066±0.00Dd	0.0046±0.00Dd	0.0020±0.00Cc
S1	0.0126±0.00Aa	0.0095±0.00Aa	0.0031±0.00Aa
S2	0.0094±0.00Bb	0.0066±0.00Bb	0.0028±0.00AaBb
S3	0.0078±0.00Cc	0.0052±0.00Cc	0.0026±0.00Bb
S4	0.0059±0.00Dd	0.0039±0.00Dd	0.0020±0.00Cc

Note: Content is presented as mean ± standard deviation. Different lowercase letters indicate significant differences in nutrient content ($p < 0.05$), and different uppercase letters indicate extremely significant differences in nutrient content ($p < 0.01$).

Table S8 Calibration curve equations

Compound	Equations	R^2
Citric acid	y = 5.6206x - 0.0065	1
Malic acid	y = 4.0368x - 0.0173	0.9999
Chlorogenic acid	y = 4E+06x - 5106.8	0.9989
Rutin	y = 4E+06x - 5106.8	0.9999
Amygdalin	y = 0.1143x + 0.3462	0.9996
